# 双根超细胸腔引流管在单孔胸腔镜肺部手术中的应用

**DOI:** 10.3779/j.issn.1009-3419.2021.101.23

**Published:** 2021-08-20

**Authors:** 磊 陈, 勇兵 陈, 雪杰 吴, 星 金, 雪娟 朱

**Affiliations:** 215004 苏州，苏州大学附属第二医院胸心外科 Department of Cardiothoracic Surgery, the Second Affiliated Hospital, Soochow University, Suzhou 215004, China

**Keywords:** 单孔胸腔镜手术, 肺部疾病, 超细胸腔引流管, Uniportal video-assisted thoracoscopic surgery, Pulmonary disease, Ultrafine chest tube

## Abstract

**背景与目的:**

目前在单孔胸腔镜肺手术中需留置双根胸腔引流管时常联合使用细管与粗管，且粗管多置于切口内，增加了术后疼痛感及切口愈合不良风险。本研究将评估单孔胸腔镜肺部术后采用双根10 F超细猪尾巴管引流的疗效及安全性。

**方法:**

回顾苏州大学附属第二医院胸心外科同一治疗组2018年6月-2020年6月的单孔胸腔镜肺手术病历资料，对比在不同时期分别采用“10 F超细猪尾巴管+24 F粗管”及“双根10 F超细猪尾巴管”两种胸腔引流方案的效果。

**结果:**

2019年6月及以后采用“双根10 F超细猪尾巴管”方案的A组共有106例，2019年6月及以前采用10 F超细猪尾巴管+24 F粗管的B组共有183例。术后A、B两组胸腔引流液量(mL)(1^st^: 199.54±126.56 *vs* 203.59±139.32, *P*=0.84; 2^nd^: 340.30±205.47 *vs* 349.74±230.92, *P*=0.76; 3^rd^: 435.19±311.51 *vs* 451.37±317.03, *P*=0.70; 4^th^: 492.58±377.33 *vs* 512.57±382.94, *P*=0.69; 共计: 604.57±547.24 *vs* 614.64±546.08, *P*=0.88)、引流管留置时间(d)(上管：2.54±2.20 *vs* 3.40±2.07, *P*=0.21;下管：2.24±2.43 *vs* 3.82±2.12，*P*=0.10)、术后住院时间(d)(6.87±3.17 *vs* 7.06±3.21, *P*=0.63)、切口愈合不良情况(0 *vs* 3.28%, *P*=0.09)、术后调整下胸腔引流管情况(0.94% *vs* 2.19%, *P*=0.66)、术后第一次视觉模拟量表(visual analogue score, VAS)(3.00±0.24 *vs* 2.99±0.15, *P*=0.63)在两组之间均无统计学差异。但A组术后VAS_2_(2.28±0.63 *vs* 2.92±0.59, *P* < 0.01)、VAS_3_(2.50±1.58 *vs* 2.79±1.53, *P*=0.02)、术后追加镇痛药物频次(25.47% *vs* 38.25%, *P*=0.03)及术后调整上胸腔引流管的频次(0 *vs* 4.37%, *P*=0.03)均较B组显著偏低。

**结论:**

在部分高选择的单孔胸腔镜肺手术过程中采用双根10 F超细猪尾巴管引流安全有效，可减少术后疼痛，降低术后胸腔引流管重置发生率。

## Abstract (en)

**Background and objective:**

At present, an ultrafine chest tube combined with a traditional thick tube were often used after pulmonary uniportal video-assisted thoracoscopic surgery (U-VATS). However, the thick tube was often placed in the incision, which increased the risk of poor wound healing and postoperative pain. The aim of this study is to investigate the feasibility and safety of using two ultrafine chest tubes (10 F pigtail tube) for drainage after pulmonary U-VATS.

**Methods:**

The medical records of patients who underwent pulmonary U-VATS during June 2018 and June 2020 in the department of cardiothoracic surgery of the second affiliated hospital of Soochow university were retrospectively reviewed to compare two different drainage strategies, receiving two 10 F pigtail tubes as chest tube (group A) or one 10 F pigtail tube as lower chest tube combined with one 24 F tube as upper chest tube (group B).

**Results:**

106 patients in group A receiving two 10 F pigtail tubes during June 2019 and June 2020 and 183 patients in group B receiving one 10 F pigtail tube as lower chest tube combined with one 24 F tube as upper chest tube during June 2018 and June 2019 were included. There was no significant difference between two groups in terms of the postoperative thoracic drainage (mL) (1^st^: 199.54±126.56 *vs* 203.59±139.32, *P*=0.84; 2^nd^: 340.30±205.47 *vs* 349.74±230.92, *P*=0.76; 3^rd^: 435.19±311.51 *vs* 451.37±317.03, *P*=0.70; 4^th^: 492.58±377.33 *vs* 512.57±382.94, *P*=0.69; Total: 604.57±547.24 *vs* 614.64±546.08, *P*=0.88), drainage time (d) (upper chest tube: 2.54±2.20 *vs* 3.40±2.07, *P*=0.21; lower chest tube: (2.24±2.43 *vs* 3.82±2.12, *P*=0.10), postoperative hospital stays (d) (6.87±3.17 *vs* 7.06±3.21, *P*=0.63), poor wound healing (0 *vs* 3.28%, *P*=0.09), replacement of lower chest tube (0.94% *vs* 2.19%, *P*=0.66) and the VAS_1_ (3.00±0.24 *vs* 2.99±0.15, *P*=0.63). Notably, there were significant differences between two groups in terms of the VAS_2_ (2.28±0.63 *vs* 2.92±0.59, *P* < 0.01) and VAS_3_ (2.50±1.58 *vs* 2.79±1.53, *P*=0.02), as well as the frequency of using additional analgesics (25.47% *vs* 38.25%, *P*=0.03) and replacement of the upper chest tube (0 *vs* 4.37%, *P*=0.03).

**Conclusion:**

It's feasible and safe to use two 10 F pigtail tubes for drainage after pulmonary U-VATS, which can achieve less postoperative pain and lower frequency of replacement of the upper chest tube on some specific patients.

近年来，随着单孔胸腔镜技术快速发展，手术切口越来越小，术中留置胸腔引流管的方法和镇痛方法也在持续改进。在单孔胸腔镜肺手术中根据术中情况，可不使用引流管、使用单根引流管或联合使用细管和粗管的方案^[1-5]^。双根胸管方案多采用经肋间穿刺置入一根细管联合切口内留置一根16 F-28 F粗管的方式。然而切口内留置引流管，尤其是需较长时间保留将大大增加术后疼痛及切口愈合不良的风险。本研究将我中心同一治疗组在2018年6月-2020年6月进行的单孔胸腔镜肺手术中采用的双根10 F超细猪尾巴管引流，或24 F粗管联合1 0F超细猪尾巴管引流两种不同的方案进行对比分析。

## 1 资料与方法

### 1.1 一般资料

苏州大学附属第二医院胸心外科2018年6月-2019年6月之间单孔胸腔镜肺手术中部分需要留置双根胸腔引流管的患者采用“10 F猪尾巴管+24 F粗管”方案，而2019年6月以后则采用“双根10 F猪尾巴管”方案，其中2019年6月为两种引流方案的过渡时期。本研究纳入2018年6月-2020年6月我科同一治疗组收治的肺部原发性或转移性恶性肿瘤、部分良性肺肿瘤以及肺脓肿、支气管扩张伴咯血、气胸等良性病变病例。收集2019年6月及以后采用“双根10 F猪尾巴管”方案的病例资料(A组)以及2019年6月及以前采用的“10 F猪尾巴管+24 F粗管”方案的病例资料(B组)。纳入标准：①单孔胸腔镜肺手术; ②术中经评估后留置双根胸腔引流管。排除标准：①全胸膜腔严重广泛粘连; ②术中或术后合并大出血; ③术后合并支气管胸膜瘘; ④术后严重低蛋白血症或乳糜胸合并大量胸腔积液; ⑤术后意外拔管。本回顾性临床研究方案已通过苏州大学附属第二医院伦理委员会审批。

### 1.2 治疗方法

#### 1.2.1 手术方式

根据术中漏气、渗血情况以及术式，部分患者不需留置胸腔引流管，部分患者仅需置入单根或需要留置双根胸腔引流管。较严重的漏气或渗血肺切缘予以4-0聚丙烯线连续缝合。本研究中肺段、肺叶切除手术患者、多个位置部分切除手术患者以及单一位置肺部分切除的特殊患者，如支气管扩张、肺脓肿、以及其他合并较严重胸腔粘连的患者均采用双根引流管方案。手术切口取患侧腋前线和腋中线之间的第4或第5肋间，长3 cm-4 cm。双细管组(A组)采用两根10 F猪尾巴超细管(图 1A)，术中上管经第2肋间锁骨中线稍偏外侧处穿刺置入，引流管位于肺组织前方，螺旋侧孔部分的平面与前胸廓内侧切面平行(图 1B); 下管根据膈肌高低在第7或8肋间腋中线到腋后线之间，稍斜向后上方穿刺置入，螺旋侧孔部分位于肺与膈肌之间偏后纵隔处，其平面平行贴附于膈肌表面(图 1C); 切口内无引流管，并采用可吸收线皮内缝合。细管+粗管组(B组)上管采用一根24 F粗管经切口内置入胸顶部，下管置入方式同A组; 切口采用间断缝合，置管处预留一根缝线待拔管时打结用。

**图 1:**
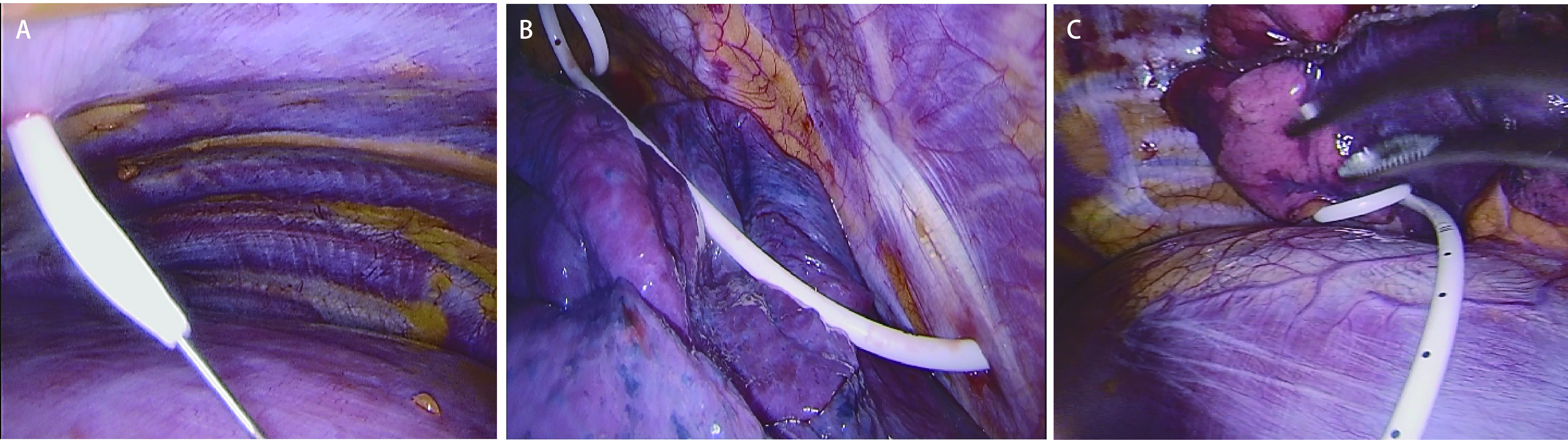
双根10 F猪尾巴超细胸腔引流管置入。A：经肋间穿刺在导丝引导下置入10 F猪尾巴管; B：置入胸顶部的上胸腔引流管; C：置入肋膈角处的下胸腔引流管。 Placement of two ultrafine chest tubes (10 F pigtail tube) for drainage. A: The guidewire was inserted into the chest as a seeker to place the pigtail tube; B: The upper drainage tube was placed on top of thorax; C: The lower drainage tube was placed at costophrenic angle.

#### 1.2.2 胸管拔除

术后第1天胸片提示肺复张良好且胸管无漏气可拔除上管; 经相关治疗好转后的术后漏气者，在夹管24 h后肺复张良好且再次开放胸管后无明显漏气者可拔除上管。连续2 d内引流液量 < 150 mL，胸水呈淡血性或淡黄色状态可拔除下管。

#### 1.2.3 疼痛管理

采用术中竖脊肌神经阻滞+术后静脉自控镇痛泵方案。术后使用视觉模拟评分法(visual analogue scale, VAS)进行疼痛评分，床位护士在患者返回病房后每8 h评估一次，连续评估3次(VAS_1_, VAS_2_, VAS_3_)，患者主诉难以忍受疼痛时(VAS≥4分)，可即刻采用自控镇痛泵追加一次或予以肌肉注射盐酸布桂嗪注射液(50 mg-100 mg)一次。

#### 1.2.4 胸管调整

术后合并轻度以上漏气者，排除活动性出血风险后上管接负压引流瓶进行负压吸引，若负压吸引后大量漏气无好转，或仍合并严重皮下气肿、肺组织明显压缩者，拔除原排气管并根据胸部计算机断层扫描(computed tomography, CT)情况重新置胸腔引流管。术后复查提示胸腔积液引流不畅或存在包裹性积液者，根据CT或胸腔B超结果调整下管位置，调整无效则行穿刺抽液或重置引流管。

### 1.3 统计学方法

采用SSPS 26.0统计学软件对数据进行分析，计量资料采用均数±标准差(Mean±SD)表示，组间比较采用独立样本的*t*检验; 计数资料两组间比较采用*χ*^2^检验、连续性校正的*χ*^2^检验或*Fisher*确切概率法检验。*P* < 0.05为差异有统计学意义。

## 2 结果

本研究共有289例病例纳入(A组106例，B组183例)。年龄、性别、手术部位、手术方案以及术后病理类型的分布等一般资料在两组之间均无统计学差异(*P* > 0.05)(表 1)。术后4 d内累计胸腔引流液以及术后胸腔总引流液量、引流管拔除时间以及术后住院时间在两组之间均无统计学差异(*P* > 0.05)(表 2)。术后疼痛评估中(表 2)，两组VAS_1_(3.00±0.24 *vs* 2.99±0.15, *P*=0.63)无统计学差异，但A组中VAS_2_(2.28±0.63 *vs* 2.92±0.59, *P* < 0.01)、VAS_3_(2.50±1.58 *vs* 2.79±1.53, *P*=0.02)以及术后追加镇痛药物情况(25.47% *vs* 38.25%, *P*=0.03)显著优于B组。B组中术后重新调整上管的频次显著多于A组(4.37% *vs* 0, *P*=0.03)(表 2)，但A、B两组之间下管调整频次(0.94% *vs* 2.19%, *P*=0.66)及切口愈合不良(0 *vs* 3.28%, *P*=0.09)情况均无统计学差异; A组患者切口采用普通可吸收线行皮内缝合后愈合佳、美观且不需拆线。

**表 1:** 两组病例一般临床资料比较 Comparison of baseline characteristics between two groups

Characteristics	Group A (*n*=106)	Group B (*n*=183)	*χ*^2^ /*t*	*P*
Age (yr)	55.28±14.51	54.71±14.20	0.39	0.70
Gender (Male/Female)	45/61	86/97	0.56	0.45
Distribution of pulmonary resection^a^			4.90	0.30
RUL	38	54		
RML	5	7		
RLL	8	27		
LUL	29	45		
LLL	6	16		
Surgical site (≥2)	20	34	0.36	0.55
Operation			5.56	0.06
Segmentectomy	30	38		
Lobectomy	41	59		
Wedge resection	35	86		
Pathology			7.28	0.19
Benign	20	58		
Tis	7	14		
MIA	32	38		
IA	41	64		
SCC	3	5		
Others^b^	3	4		
^a^Patients with only one surgical site. ^b^Other pathological types include metastatic carcinoma, poorly differentiated carcinoma, and mucinous adenocarcinoma. RUL: right upper lobe; RML: right middle lobe; RLL: right lower lobe; LUL: left upper lobe; LLL: left lower lobe; Tis: tumor *in situ*; MIA: minimally invasive adenocarcinoma; IA: invasive adenocarcinoma; SCC: squamous cell cancer. Group A: receiving two 10 F pigtail tubes for drainage; Group B: receiving one 10 F pigtail tube as lower chest tube combined with one 24 F tube as upper chest tube for drainage.				

**表 2:** 两组病例术后临床资料比较 Comparison of perioperative outcomes of the included patients

Characteristics	Group A (*n*=106)	Group B (*n*=183)	*χ*^2^ /*t*	*P*
Accumulative thoracic drainage (mL)^a^				
1^st^	199.54±126.56	203.59±139.32	-0.20	0.84
2^nd^	340.30±205.47	349.74±230.92	-0.31	0.76
3^rd^	435.19±311.51	451.37±317.03	-0.38	0.70
4^th^	492.58±377.33	512.57±382.94	-0.40	0.69
Total	604.57±547.24	614.64±546.08	-0.15	0.88
Drainage time (d)				
Upper chest tube	2.54±2.20	3.40±2.07	1.25	0.21
Lower chest tube	2.24±2.43	3.82±2.12	-1.67	0.10
Postoperative VASb				
VAS_1_	3.00±0.24	2.99±0.15	0.48	0.63
VAS_2_	2.28±0.63	2.92±0.59	-8.70	0.00
VAS_3_	2.50±1.58	2.79±1.53	-2.28	0.02
Additional analgesics (times)	27	70	4.92	0.03
Replacement of the chest tube (times)				
Upper chest tube	0	8	4.77	0.03
Lower chest tube	1	4	0.61	0.66
Poor wound healing	0	6	3.55	0.09
Postoperative hospital stays (d)	6.87±3.17	7.06±3.21	-0.48	0.63
^a^1^st^: The cumulative thoracic drainage within 1 d after operation; 2^nd^: The cumulative thoracic drainage within 2 d after operation; 3^rd^: The cumulative thoracic drainage within 3 d after operation; 4^th^: The cumulative thoracic drainage within 4 d after operation; ^b^Postoperative pain scores were performed using visual analogue scale (VAS), and the nurses evaluated VAS every 8 h after surgery. VAS_1_: VAS of the first 8 h after surgery; VAS_2_: VAS of the second 8 h after surgery; VAS_3_: VAS of the third 8 h after surgery.				

## 3 讨论

加速康复外科(enhanced recovery after surgery, ERAS)在胸外科的运用不仅在于呼吸道管理、营养管理及活动锻炼等方面，也在于微创手术方案选择及疼痛管理^[6]^。术后疼痛主要源于手术创伤及引流管刺激。由于剧烈的疼痛使得患者术后不敢咳嗽排痰，不愿早期下床活动，从而大大增加了肺不张及肺部感染风险。因此，引流管方案对降低术后疼痛、促进术后快速康复具有重要意义^[7-9]^。目前术后引流方案有：经肋间穿刺置入细管+经切口内置入粗管(16 F-28 F)^[2, 10]^以及部分病例不置胸管或单根胸管^[4, 5]^。这些方案较传统28 F或32 F粗管能有效地减轻疼痛，也验证了穿刺置入细管引流安全有效^[2, 3]^。

在本中心，我们根据术中情况，对部分简单的亚肺叶切除患者不置管或置单根胸管，其他患者则采用双根引流管。但我们早期采用的切口内留置16 F-28 F引流管的传统方法，仍会增加疼痛刺激及切口愈合不良风险。因此，我们在部分需要置入双根胸腔引流管的患者中，探索使用双根10 F超细猪尾巴管经肋间穿刺置入的引流方案。我们发现A、B两组术后胸腔引流液量、上下胸腔引流管的留置时间、术后住院时间均无明显统计学差异。说明单孔胸腔镜肺手术过程中，部分患者采用双根10 F猪尾巴管引流与传统方式相比同样安全有效。

由于本研究中绝大部分患者在术后第1天已拔出排气管，因此研究中采集的VAS是术后每8 h评估一次，连续评估3次，遇特殊疼痛发作时加评。两组术后3次VAS中VAS_1_均为最高(3.00±0.24 *vs* 2.99±0.15, *P*=0.63)，考虑与术后早期手术创伤的急性疼痛刺激相关，但由于术后麻醉镇痛药物代谢周期存在，且患者早期静卧，故两组之间并无明显统计学差异。VAS_2_评估时患者已处于术后第一天但尚未拔管，术后急性疼痛刺激已明显减退，从而两组VAS_2_虽均稍有下降; 但患者目前已开始进行一般活动及咳嗽排痰行为，由于细管疼痛刺激小，故A组VAS_2_较B组偏低(2.28±0.63 *vs* 2.92±0.59, *P* < 0.01)。VAS_3_评估时已开始进行更多术后活动、拍背及自主咳嗽排痰行为，且处于胸管拔管前后状态，此时A组VAS_3_同样较B组显著偏低(2.50±1.58 *vs* 2.79±1.53, *P*=0.02)。因此，我们认为经肋间穿刺置入细管较经切口内置入粗管疼痛刺激更小。

另外，胸腔镜手术的单孔位置大多处于第4或第5肋间腋前线与腋中线之间，部分切口会靠近腋中线位置，24 F粗管均从切口内置入。在遭遇术后中度漏气需长期留置粗管时，切口愈合不良的风险以及疼痛刺激增加，甚至部分引流管位置不佳的中度漏气患者以及几乎所有重度漏气的患者都需要在近锁骨中线第二肋间处重置粗胸管。然而，猪尾巴管在术中直接放置在近锁骨中线第二肋间的传统排气管位置，因此在长期漏气患者中，依靠调节负压强度可达到充分排气作用，从而减少了重置胸腔引流管的二次创伤，降低了切口愈合不良风险及疼痛刺激。因此，术后发生较严重漏气且需长期置管的患者，采用猪尾巴管作为上管较少需要重置胸管(0 *vs* 4.37%, *P*=0.03)。然而，两组采用猪尾巴管作为下管引流效果均佳，且重置情况无明显差异(0.94% *vs* 2.19%, *P*=0.66)。

虽然A、B两组切口愈合不良情况并无统计学差异(0 *vs* 3.28%, *P*=0.09)，但采用可吸收线行皮内缝合后手术疤痕美观且免拆线，满足青年化手术人群的美观需求。而愈合不良发生率差异不明显可能与B组术后发生漏气且排气欠佳的病例被极早拔除胸管相关。我们将切口内胸管拔除后置换至锁骨中线第2肋间处，及时消除了切口内长期留置引流管引起切口愈合不良的高危因素，但不可避免地增加了术后二次创伤。

研究中由于病种类型较多，不同疾病术后漏气和渗血风险不一，可能对引流液量及置管时间分析产生影响; 另外术后第一根引流管拔除的时间节点与VAS_3_评估时间节点相近，其先后关系会影响到VAS分值，而本中心VAS_3_以后的评估数据仅针对于部分急性疼痛发作患者，不足以进行有效的分析，有待进一步研究。

综上，我们认为在部分高选择的单孔胸腔镜肺手术过程中采用双根10 F超细猪尾巴管引流的方案安全有效，同时可减少部分患者术后疼痛、降低术后胸腔引流管重置发生率。从目前临床上手术周转较快，且患者对术后快速康复要求较高的实际情况出发，减少疼痛及降低术后二次创伤具有较重要的临床意义。
